# *In vitro* evaluation of periapical lesion‐derived stem cells for dental pulp tissue engineering

**DOI:** 10.1002/2211-5463.13336

**Published:** 2021-12-05

**Authors:** Weiping Li, Mengying Mao, Nan Hu, Jia Wang, Jing Huang, Shensheng Gu

**Affiliations:** ^1^ Department of Endodontics and Operative Dentistry Shanghai Ninth People’s Hospital College of Stomatology Shanghai Jiao Tong University School of Medicine Shanghai China; ^2^ Department of Oral and Maxillofacial Head & Neck Oncology Shanghai Ninth People's Hospital College of Stomatology Shanghai Jiao Tong University School of Medicine Shanghai China; ^3^ National Center for Stomatology National Clinical Research Center for Oral Diseases Shanghai Key Laboratory of Stomatology Shanghai China

**Keywords:** dental pulp regeneration, immunomodulatory, odontogenesis, osteogenesis, periapical lesion‐derived stem cells, pro‐angiogenesis

## Abstract

Dental pulp tissue engineering is a promising alternative treatment for pulpitis and periapical periodontitis, and dental pulp stem cells (DPSCs) are considered to be the gold standard for dental seed cell research. Periapical lesions harbor mesenchymal stem cells with the capacity for self‐renewal and multilineage differentiation. However, it remains unknown whether these periapical lesion‐derived stem cells (PLDSCs) are suitable for dental pulp tissue engineering. To investigate this possibility, PLDSCs and DPSCs were isolated using the tissue outgrowth method and cultured under identical conditions. We then performed *in vitro* experiments to investigate their biological characteristics. Our results indicate that PLDSCs proliferate actively *in vitro* and exhibit similar morphology, immunophenotype and multilineage differentiation ability as DPSCs. Simultaneously, PLDSCs exhibit stronger migrative ability and express more vascular endothelial growth factor and glial cell line‐derived neurotrophic factor than DPSCs, and PLDSC‐derived conditioned medium was more effective in tube formation assay. The mRNA expression levels of immunomodulatory genes *HLA‐G*, *IDO* and *ICAM‐1* were also higher in PLDSCs. However, regarding osteo/odontogenic differentiation, PLDSCs showed weaker alkaline phosphatase staining and lower calcified nodule formation compared to DPSCs, as well as lower expression of *ALP*, *RUNX2* and *DSPP*, as confirmed by a quantitative RT‐PCR. The osteo/odontogenic protein expression levels of DSPP, RUNX2, DMP1 and SP7 were also higher in DPSCs. The present study demonstrates that PLDSCs demonstrate potential use as seed cells for dental pulp regeneration, especially for achieving enhanced neurovascularization.

## Graphical abstract

Stem cells from periapical lesions have not previously been investigated in the context of dental pulp tissue engineering. In the present study, we compared periapical lesion‐derived stem cells (PLDSCs) with dental pulp stem cells (DPSCs) *in vitro*. We found that PLDSCs have greater migrative, angiogenic, neurotrophic and immunomodulatory effects, but weaker proliferative and osteo/odontogenic ability, compared to DPSCs.

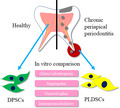

**Abbreviations**
ALP: alkaline phosphatase
CCK‐8: Cell Counting Kit‐8
CFU‐F: colony‐forming units‐fibroblasts
DMEM: Dulbecco’s modified Eagle’s medium
DMP1: dentin matrix protein 1
DPSCs: dental pulp stem cells
DSPP: dentin sialophosphoprotein
GDNF: glial cell line‐derived neurotrophic factor
HGF: hepatocyte growth factor
HLA‐G: human leukocyte antigen G
HUVECs: human umbilical vein endothelial cells
ICAM‐1: intercellular adhesive molecule‐1
IDO: indoleamine 2,3‐dioxygenase
MSCs: mesenchymal stem cells
PLDSCs: periapical lesion‐derived stem cells
qRT‐PCR: quantitative RT‐PCR
RUNX2: RUNX family transcription factor 2
SP7: Sp7 transcription factor
VEGF: vascular endothelial growth factor

Recent advances in the field of dental pulp regenerative engineering have resulted in bench‐to‐bedside treatments [1, 2]. Mesenchymal stem cells (MSCs), especially oral‐derived MSCs, are promising tools in dental pulp tissue engineering as a result of their readily accessibility. Among them, dental pulp stem cells (DPSCs) were first characterized by Gronthos *et al*. [3] and are still the most frequently studied type of cells, being considered as the gold standard for dental seed cells research. Subsequently, a variety of dental MSCs, including stem cells from human exfoliated deciduous teeth [4], stem cells from apical papilla [5], dental follicle stem cells [6] and periodontal ligament stem cells [7], have been cultivated from the oral cavity. Nevertheless, it is still imperative to find alternative sources of seed cells as a result of the vulnerability of MSCs *in vitro*, difficulty of acquiring a sufficient number of well‐functioning cells and ethical problems pertaining to the cultivation of these cells.

Following the discovery of the immunomodulatory ability of MSCs, it has been reported that inflammation not only has little impact on the survival of MSCs, but also can active *de novo* MSCs as well as attract MSCs from other sites to contribute to the healing process [8]. Furthermore, it was also reported that MSCs from inflammatory conditions varied in characters such as proliferation, migration, differentiation potential, growth factors secretion and immunomodulatory ability [9, 10, 11]. Periapical periodontitis, a consequence of the inflammatory response to the pulp necrosis and bacterial invasion, was recently found to be a potential source of cells exhibiting MSC properties [12, 13]. Therefore, the pathological tissues that were once thrown away have now been recognized as a rich source of MSCs. However, these periapical lesion‐derived stem cells (PLDSCs) have not been investigated in the context of dental pulp regeneration.

For use in dental pulp regeneration, the pro‐angiogenesis ability plays a pivotal role because the dentin–pulp complex is nourished only by few blood vessels entering through the narrow root canal apex [14]. The pro‐angiogenesis ability of dental MSCs has already been confirmed in many studies [14, 15]. Therefore, as oral‐derived stem cells, PLDSCs might act as promising pro‐angiogenesis seed cells for dental pulp engineering. Furthermore, osteo/odontogenic differentiation ability and neurogenesis induced ability are also required for seed cells to achieved complete dentin–pulp complex regeneration. However, whether the characteristics of PLDSCs are suitable for dental pulp engineering remain unknown. To clarify this issue, we cultured PLDSCs and DPSCs under identical condition and compared multiple aspects of them.

## Materials and methods

### Cell isolation and culture

Patients (*n* = 3) aged 18–25 years and with chronic periapical periodontitis were informed and had signed written informed consent in the study. All subsequent protocols were approved by the Ethical Committee of Shanghai Ninth People’s Hospital. The study methodologies conformed to the standards set by the Declaration of Helsinki. Before microscopic endodontic surgery, complete blood counts, coagulation function and the presence of infectious diseases were examined. In addition, all patients underwent periodontal treatment 1 week prior to surgery. The surgeon and surgical protocols remained the same for all enrolled subjects. During the surgery, the removed inflamed tissue was placed in sterile phosphate‐buffered saline (PBS) and then transferred to the laboratory within 10 min. The whole tissue was then washed several times with sterile PBS containing 100 U·mL^−1^ penicillin and 100 mg·mL^−1^ streptomycin (Invitrogen, Waltham, MA, USA) until the mixture became transparent. Then, the tissue outgrowth method was applied. Briefly, the tissue was placed in a 10‐mm dish for mechanical disruption. The samples were then minced into small pieces (approximately 0.1 mm in diameter) using sterile ophthalmic scissors. The minced tissues were transferred to 10‐mm culture dishes, followed by the application of sterile cover glasses dipped in sterile petroleum jelly to the four corners for tissue stability. High‐glucose Dulbecco’s modified Eagle’s medium (DMEM (Gibco, Walthem, MA, USA) supplemented with 10% FBS (Gibco), 100 U·mL^−1^ penicillin and 100 mg·mL^−1^ streptomycin (Invitrogen) was used to culture the cells. The culture dishes were incubated at 37 °C and 5% CO_2_, and the medium was changed every 3 days. The cells were passaged when they reached about 100% confluence. To isolate DPSCs, premolars or wisdom teeth without cavities for orthodontic reasons were extracted from healthy patients aged 18–25 years (*n* = 3), followed by gentle separation of the dental pulp. The culturing steps were similar to those for PLDSCs. To acquire an objective baseline, we used passage 3 of both cell types to perform the following experiments.

### Flow cytometry assay

DPSCs and PLDSCs were identified via the cell surface antigens CD45, CD31, CD34, CD44, CD29, CD73, CD90 and CD105 using a flow cytometry assay. Cell were fixed in 4% phosphate‐buffered paraformaldehyde and incubated with CD45‐FITC (Invitrogen), CD31‐PE (Becton Dickinson, Franklin Lakes, NJ, USA), CD34‐PE (BD Bioscience, San Jose, CA, USA), CD44‐FITC (Invitrogen), CD29‐PE (Invitrogen), CD73‐PE (eBioscience, San Diego, CA, USA), CD90‐FITC (eBioscience) and CD105‐PE (eBioscience) antibodies for 45 min. Then, the cells were washed twice with PBS and analyzed via a FACSCalibur flow cytometer (Becton Dickinson).

### Immunocytochemistry

To detect the expression of vascular endothelial growth factor (VEGF) and glial cell line‐derived neurotrophic factor (GDNF), a sterile round cover glass for cell growth was placed into a 24‐well plate. DPSCs and PLDSCs were seeded at a density of 2.5 × 10^4^ cells·mL^−1^ onto the cover glass. After culturing the cells for 3 days with growth medium (DMEM supplemented with 10% FBS, 100 U·mL^−1^ penicillin and 100 mg mL^−1^ streptomycin), they were fixed with 4% paraformaldehyde for 20 min. Cells were permeabilized using 1% Triton X‐100 and 3% BSA was used to block non‐specific binding. Then, the samples were incubated with primary antibodies against VEGF (dilution 1 : 100; MA5‐13182; Thermo Fisher, Waltham, MA, USA) and GDNF (dilution 1 : 200; #711074; Thermo Fisher), Alexa Fluor 555‐labeled donkey anti‐mouse IgG secondary antibodies (dilution 1 : 500; A0460; Beyotime, Shanghai, China) and Alexa Fluor 555‐labeled donkey anti‐rabbit IgG secondary antibodies (dilution 1 : 500; A0453; Beyotime) was then applied respectively. The cytoskeleton was stained with fluorescein isothiocyanate–phalloidin (dilution 1 : 200; #40735ES75; Yeasen, Shanghai, China) for 1 h at room temperature, followed by the addition of 4′,6‐diamidino‐2‐phenylindole (C1005; Beyotime) to stain the nuclei. Images were acquired using a confocal laser scanning microscope (Leica, Wetzlar, Germany).

### *In vitro* osteo/odontogenic differentiation

Both cell types were detached with 0.25% trypsin–EDTA, resuspended in growth medium and seeded at a density of 10 × 10^4^ cells·well^−1^ into a 24‐well plate. After reaching 90% confluence, the medium was replaced with osteo/odontogenic medium containing 10% FBS, 0.2 mm l‐ascorbic acid‐2‐phosphate, 100 nm dexamethasone, 10 mm β‐glycerophosphate, 100 U·mL^−1^ penicillin and 100 mg·mL^−1^ streptomycin. The medium was changed every 3 days. After culturing the cells for 7, 14, 21 and 28 days, they were washed three times with PBS and fixed with 4% paraformaldehyde for 20 min. A 1% Alizarin Red S (A5533; Sigma‐Aldrich, St Lois, MO, USA) solution, dissolved in isopropanol and filtered through a 0.22‐μm filter, was added at a volume of 1 mL·well^−1^ for 15 min to detect the presence of calcified nodules. Then, the samples were washed with PBS until the water became transparent, followed by observation under an inverted phase‐contrast microscope and simultaneous imaging.

### *In vitro* adipogenic differentiation

To assess the adipogenic differentiation capacity of PLDSCs and DPSCs, cells were incubated in adipogenic differentiation medium kit (HUXDP‐90031; Cyagen, Santa Clara, CA, USA) supplemented with 10% FBS, 100 U·mL^−1^ penicillin, 10 μg·mL^−1^ streptomycin, 12 mm l‐glutamine, 10 μm insulin, 200 μm indomethacin, 1 μm dexamethasone and 0.5 mm 3‐isobutyl‐1‐methylxanthine. After culturing the cells for 3 weeks, they were fixed and stained with oil red O stain for 30 min. Images were captured under an inverted phase‐contrast microscope.

### *In vitro* chondrogenic differentiation

To investigate the chondrogenic differentiation capacity of PLDSCs and DPSCs, a chondrogenic differentiation medium kit (HUXMA‐90041; Cyagen) was applied in accordance with the manufacturer’s instructions. In brief, 4 × 10^4^ cells were transferred to a centrifuge tube and resuspended with chondrogenic differentiation medium and cultured for 24 h. When the cells aggregated, the medium was changed gently with fresh chondrogenic differentiation medium. After 28 days of induction, the cell aggregates were fixed and embedded in paraffin, and cross‐sections were stained with Alcian blue. Images were then captured using an optical microscope.

### Cell proliferation assay

A Cell Counting Kit‐8 (CCK‐8) (Dojindo, Shanghai, China) assay was used to assess proliferation. DPSCs and PLDSCs were digested with 0.25% trypsin–EDTA, resuspended in culture medium and then seeded into a 96‐well plate at 5000 cells·well^−1^ with five replicate wells each. All cells were cultured in DMEM for 1, 3, 5, 7, 9 and 11 days. At each time point, CCK‐8 solution (dilution 1 : 100 with serum‐free DMEM) was added into the wells and incubated for 1 h at 37 °C. After incubation, attenuance was determined at 450 nm. To compare the proliferation ability of the cells, relative attenuance values were used for normalization and proliferation curves were plotted.

### Colony‐forming units‐fibroblasts (CFU‐F) assay

The cells were digested and seeded in a six‐well plate at a density of 1000 cells·well^−1^ in triplicate. After 14 days of culture, the cells were fixed with 4% paraformaldehyde and then stained with 1% crystal violet (C0121; Beyotime). Aggregates of more than 50 cells were scored as one colony. The number of colonies of each cell type was then counted using an inverted phase‐contrast microscope. The colony‐forming rate was determined as: colonies/1000 × 100%.

### Transwell assay

Both cell types were resuspended with serum‐free DMEM and seeded at a density of 2.5 × 10^4^ cells·mL^−1^ in the upper chamber of a 0.22‐μm transwell plate (*n* = 3). The bottom chamber was filled with DMEM supplemented with 10% FBS. After incubating the plate for 24 h, a cotton swab was used to wipe off the cells remaining on the upper chamber. Then, the cells were fixed and stained with 1% crystal violet for 15 min. The stained cells were counted under an inverted phase‐contrast microscope, with simultaneous imaging.

### Alkaline phosphatase (ALP) staining

ALP is an important enzyme involved in the early stages of osteogenesis and odontogenesis. The BCIP/NBT Alkaline Phosphatase Color Development Kit (C3206; Beyotime) was used to detect ALP expression in accordance with the manufacturer’s instructions. DPSCs and PLDSCs were seeded into 24‐well plates at a density of 2.5 × 10^4^ cells·mL^−1^ in triplicate. After reaching more than 90% confluence, the culture medium of the experimental group was exchanged with osteo/odontogenic medium containing 10% FBS, 0.2 mm l‐ascorbic acid‐2‐phosphate, 100 nm dexamethasone, 10 mm β‐glycerophosphate, 100 U·mL^−1^ penicillin and 100 mg·mL^−1^ streptomycin. After 3 and 7 days of culture, the cells were fixed and stained with ALP working solution at 37 °C for 20 min in dark condition. The reaction was stopped using PBS, and images were captured using an inverted phase‐contrast microscope.

### Quantitative RT‐PCR (qRT‐PCR)

DPSCs and PLDSCs were seeded in a six‐well plate at a density of 10 × 10^4^ cells·mL^−1^. After reaching more than 90% confluence, the culture medium in experimental groups was replaced with osteo/odontogenic medium (containing 10% FBS, 0.2 mm l‐ascorbic acid‐2‐phosphate, 100 nm dexamethasone, 10 mm β‐glycerophosphate, 100 U·mL^−1^ penicillin and 100 mg·mL^−1^ streptomycin) and cultured for 3, 7 days, while the control groups replaced with growth medium. At each time point, the total RNA of DPSCs and PLDSCs was extracted using the Trizol reagent after reaching more than 90% confluence. All RNA was then reverse‐transcribed to cDNA using a reverse transcription kit (RR036A; Takara, Beijing, China). RT‐PCR was performed using a quantitative real‐time PCR system (Roche, Basel, Switzerland) with the settings: 95 °C for 30 s for one cycle, followed by 40 cycles of 95 °C for 10 s and 60 °C for 30 s. The comparative ΔCt method was used to calculate the relative expression levels of *ALP*, *RUNX2*, *DSPP*, *VEGF*, *GDNF*, *IDO‐1*, *HLA‐G*, *HGF* and *ICAM‐1* genes. The *ACTB* gene was used for normalization. All primers were commercially synthesized (Sangon, Shanghai, China) and are listed in Table 1.

**Table 1:** Primers used in the qRT‐PCR.

Gene name	Accession number	Product length (bp)	Primer (5′‐ to 3′)	Primer sequence
VEGF	NM_001025366.3	186	Forward	TGACAGGGAAGAGGAGGAGA
			Reverse	CGTCTGACCTGGGGTAGAGA
RUNX2	NM_001015051.4	127	Forward	CACTGGCGCTGCAACAAGA
			Reverse	CATTCCGGAGCTCAGCAGAATAA
GDNF	NM_000514.4	182	Forward	CGAACTCTTGCCCCTGACCT
			Reverse	ACAGCCACGACATCCCATAAC
IDO	NM_002164.6	124	Forward	CTGTTCCTTACTGCCAACT
			Reverse	TCCATGTTCTCATAAGTCAGG
HLA‐G	NM_001363567.2	144	Forward	CTGAGATGGAAGCAGTCTT
			Reverse	GCTCCCTCCTTTTCAATCT
HGF	NM_001010931.3	184	Forward	AGACCAATGTGCTAATAGATGTA
			Reverse	GCAGTTTCTAATGTAGTCTTTGT
ICAM‐1	NM_000201.3	69	Forward	AGCTTCGTGTCCTGTATGGC
			Reverse	TTTCTGGCCACGTCCAGTTT
ACTB	NM_001101.5	186	Forward	TGGCACCCAGCACAATGAA
			Reverse	CTAAGTCATAGTCCGCCTAGAAGCA
DSPP	NM_014208.3	157	Forward	AGTGACAGCCAGAGCAAG
			Reverse	CCTATCCCATTACCAAACT
ALP	NM_001127501.4	137	Forward	CCTTGTAGCCAGGCCCATTG
			Reverse	GGACCATTCCCACGTCTTCAC

### Western blot analysis

DPSCs and PLDSCs were culturing in growth medium or osteo/odontogenic medium (containing 10% FBS, 0.2 mm l‐ascorbic acid‐2‐phosphate, 100 nm dexamethasone, 10 mm β‐glycerophosphate, 100 U·mL^−1^ penicillin and 100 mg·mL^−1^ streptomycin) for 7, 14 and 21 days. At the time point, total protein was harvested using RIPA lysis buffer (WB, 0102; Biotechwell, Shanghai, China) supplied with PMSF; the cell lysates were then centrifuged at 15 984 ***g*** for 15 min at 4 °C, and the supernatant was collected for protein analysis. The protein concentration was determined using a BCA Protein Assay (P0012S; Beyotime). Equal amounts of cell lysates were subjected to 10% SDS/PAGE and transferred to poly(vinylidene difluoride) (FFP24; Beyotime) membranes. The membranes were blocked with 5% non‐fat milk and incubated with primary antibodies against RUNX family transcription factor 2 (RUNX2) (dilution 1 : 500; #12556S; Cell Signaling Technology, Danvers, MA, USA), dentin sialophosphoprotein (DSPP) (dilution 1 : 500; sc‐73632, Santa Cruz Biotechnology, Santa Cruz, CA, USA), dentin matrix protein 1 (DMP1) (dilution 1 : 500; ab103203; Abcam, Cambridge, UK), Sp7 transcription factor (SP7) (dilution 1 : 1000; PA5‐115697; Thermo Fisher), VEGF (dilution 1 : 100; MA5‐13182; Thermo Fisher), GDNF (dilution 1 : 200; 711074; Thermo Fisher) and GAPDH (WB0197; Biotechwell) and were then incubated with HRP‐conjugated secondary antibodies (dilution 1 : 5000; Biotechwell). Finally, the protein bands were visualized using ECL Plus reagents (Biotechwell) and analyzed using imagej (https://imagej.nih.gov/ij).

### Preparation of conditioned medium

DPSCs and PLDSCs were seeded at a density of 5000–6000 cells·cm^−2^ into culture dishes. After reaching 70%–80% confluence, the cells were washed three times with PBS and then incubated with serum‐free DMEM containing 1% penicillin–streptomycin for 48 h. The supernatant was collected and centrifuged at 4 °C at 3000 ***g*** for 3 min, followed by centrifugation at 1500 ***g*** for 5 min, filtration using 0.22‐μm filters and storage at −80 °C until further use [16].

### Tube formation assay

To assess the pro‐angiogenesis ability of the cells, an *in vitro* tube formation assay was conducted. Human umbilical vein endothelial cells (HUVECs) obtained from the cell bank at the Chinese Academy of Science were seeded at a density of 20 × 10^4^ cells·mL^−1^ into 96‐well plates pre‐coated with 60 μL of growth factor reduced Matrigel (356230; BD Bioscience) in triplicate. Then, 100 μL of serum‐free DMEM (control group) and DPSC‐ or PLDSC‐conditioned medium was added. After incubation for 6 h, images of five random fields were acquired under an inverted phase‐contrast microscope and analyzed using image j.

### Statistical analysis

All experiments were performed at least three times on three different patients. When normal data distribution was confirmed, data were analyzed using Student’s *t*‐test or one‐way analysis of variance with prism, version 7.0 (GraphPad Software Inc., San Diego, CA, USA). *P* < 0.05 was considered statistically significant.

## Results

### Identification of mesenchymal stem cell properties of DPSCs and PLDSCs

**Fig. 1:**
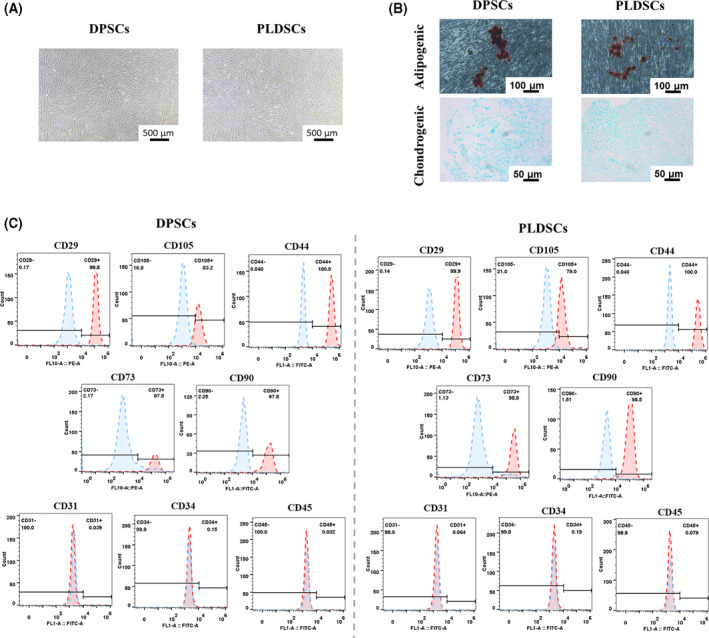
Identification of mesenchymal stem cell properties of DPSCs and PLDSCs. (A) Images obtained from phase‐contrast microscopy showing PLDSCs with a spindle‐like shape and adhesive properties similar to those of DPSCs. Scale bar = 500 μm. (B) Oil red O and Alcian blue staining showing that both DPSCs and PLDSCs can achieve adipogenic and chondrogenic differentiation. Scale bar = 100 μm (upper); 50 μm (below). (C) Immunophenotype of different cell surface markers.

Both DPSCs and PLDSCs adhered to plastic and exhibited a homogeneous spindle‐like shape from passage 1 (Fig. 1A). No obvious differences in morphology were observed. Oil red O and Alcian blue staining confirmed the adipogenic and chondrogenic differentiation of DPSCs and PLDSCs (Fig. 1B). Furthermore, the flow cytometry results showed that both DPSCs and PLDSCs expressed high levels of CD29 (> 95%), CD44 (> 95%), CD73 (> 95%), CD90 (> 95%) and CD105 (83.2% and 79%, respectively), and expressed low levels of hematopoietic marker CD31 (< 2%), CD34 (< 2%) and CD45 (< 2%) (Fig. 1C).

### Comparison of proliferative and migrative ability of PLDSCs and DPSCs

**Fig. 2:**
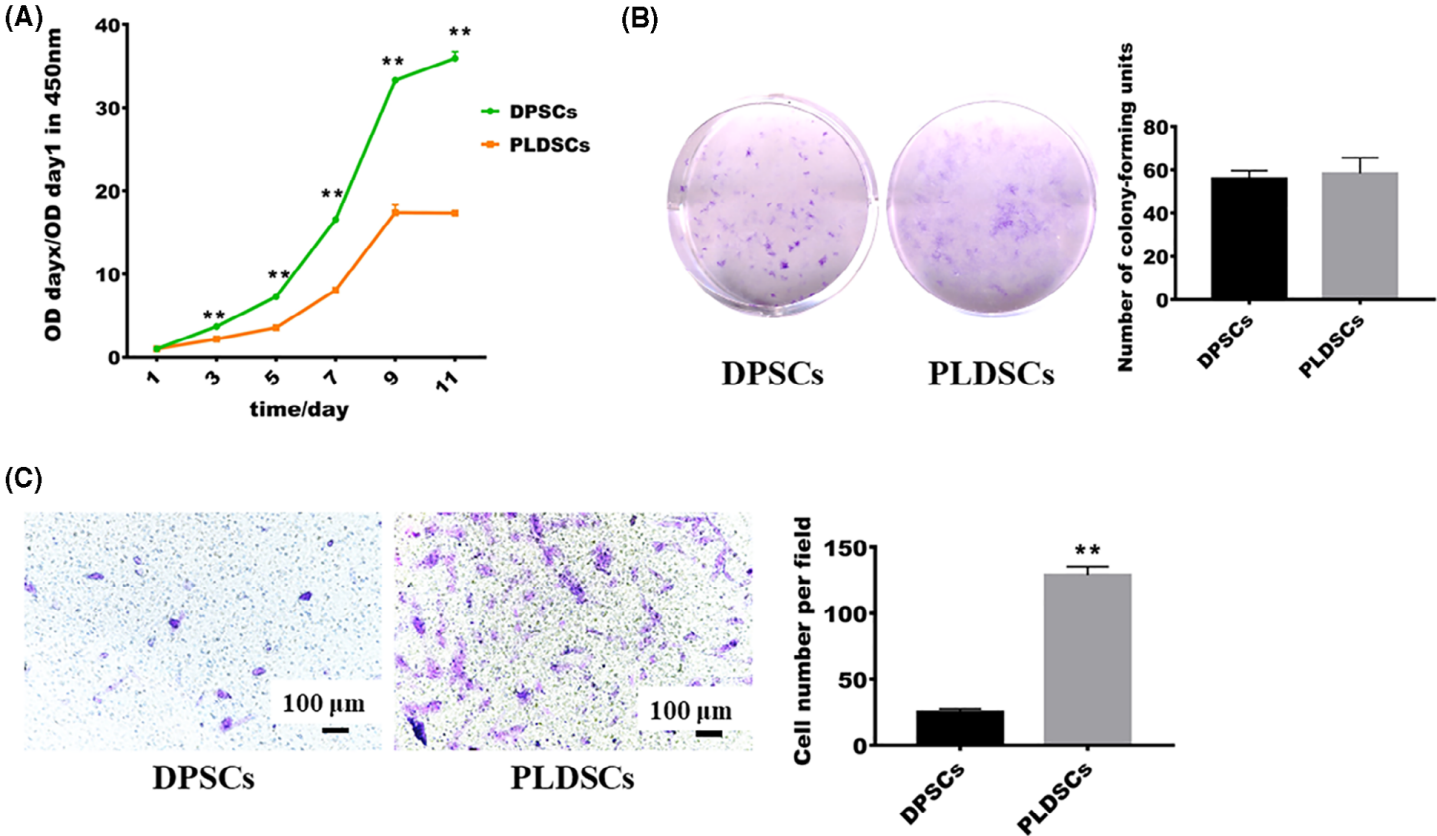
Comparison of the proliferative and migrative abilities of cells. (A) Growth curve showing the relative attenuance value from the CCK‐8 assays at different time points (*n* = 3, Student’s *t*‐test). (B) Results of the colony‐forming unit assay; there was no statistical difference between DPSCs and PLDSCs on day 14 (*n* = 3, Student’s *t*‐test). (C) Results of the transwell assay; after 24 h of culture, PLDSCs exhibited stronger migration ability (*n* = 3, Student’s *t*‐test). Scale bar = 100 μm. **P < 0.01. Data are shown as the mean ± SD.

After 3 days of culture, the relative attenuance values at 450 nm rapidly increased for DPSCs and PLDSCs, and both growth curves had a sigmoid shape, indicating that PLDSCs could be effectively expanded *in vitro*. In line with previous study [17], DPSCs had stronger proliferation ability, and both cell types entered the platform stage after 9 days of culture (Fig. 2A). The CFU‐F assay revealed that DPSCs and PLDSCs formed similar colonies after 14 days of culture and there were no significant differences between the two cell types (Fig. 2B). Considering that the stem cells suitable for tissue engineering should have migration ability, we next performed the transwell assay. After 24 h of culture, we found an increased number of migrated cells in the PLDSC group compared to the DPSC group (Fig. 2C).

### Comparison of osteo/odontogenic abilities of PLDSCs and DPSCs

**Fig. 3:**
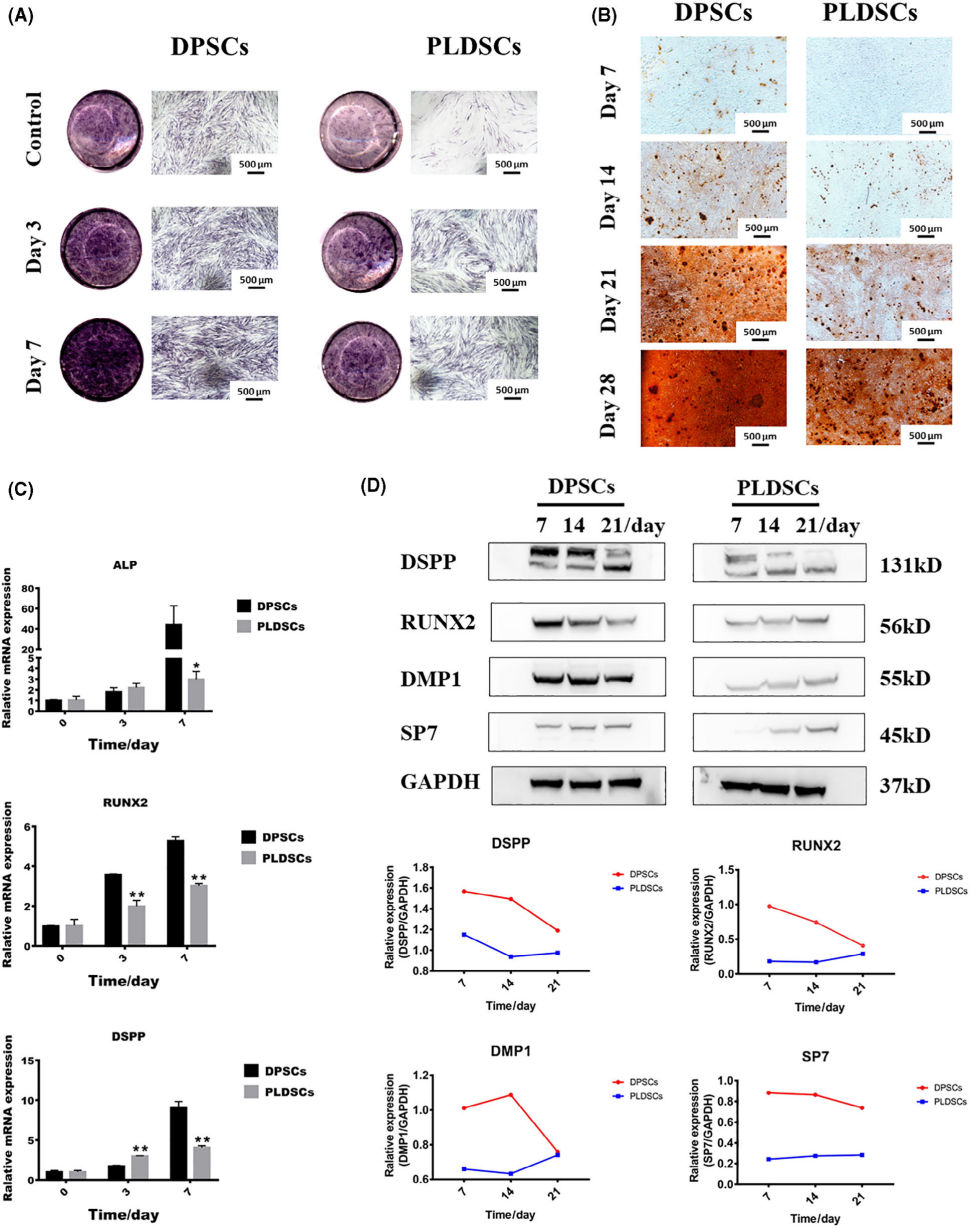
Comparison of the osteo/odontogenic capacities of the cells. (A) ALP staining after osteo/odontogenic induction for 3 and 7 days. Scale bar = 500 μm. (B) Alizarin red staining showing the presence of calcified nodules after 7, 14, 21 and 28 days of osteo/odontogenic induction. Scale bar = 500 μm. (C) Gene expression patterns of *ALP*, *RUNX2* and *DSPP* after osteogenic induction for 3 and 7 days; the expression level was normalized to that of *ACTB* (*n* = 3, Student’s *t*‐test). *P < 0.05, **P < 0.01. (D) Western blotting detection (upper) and imagej analysis (below) of DSPP, DMP1, RUNX2 and SP7 after osteogenic induction for 7, 14 and 21 days. Data are shown as the mean ± SD.

ALP is an enzyme that plays an important role in the early stages of osteogenesis and odontogenesis. After culturing in osteo/odontogenic medium for 3 and 7 days, ALP expression was enhanced in both DPSCs and PLDSCs. However, ALP expression dramatically increased in a time‐dependent manner in DPSCs, whereas only a slight increase was observed in PLDSCs (Fig. 3A). Alizarin red staining revealed that calcified nodules appeared in DPSCs after 7 days of induction, whereas there was no calcified deposition until day 14 in PLDSCs. Both DPSCs and PLDSCs could form calcified nodules by 4 weeks after induction, although there were fewer calcified nodules in PLDSCs (Fig. 3B). The qRT‐PCR results showed that mRNA expression levels of *ALP*, *RUNX2* and *DSPP* were significantly higher in DPSCs at day 7 (Fig. 3C). Western blot analysis revealed that the protein levels of DSPP, RUNX2, DMP1 and SP7 were also higher in DPSCs after 7, 14 and 21 days of osteo/odontogenic induction (Fig. 3D).

### Pro‐angiogenesis ability of PLDSCs and DPSCs

**Fig. 4:**
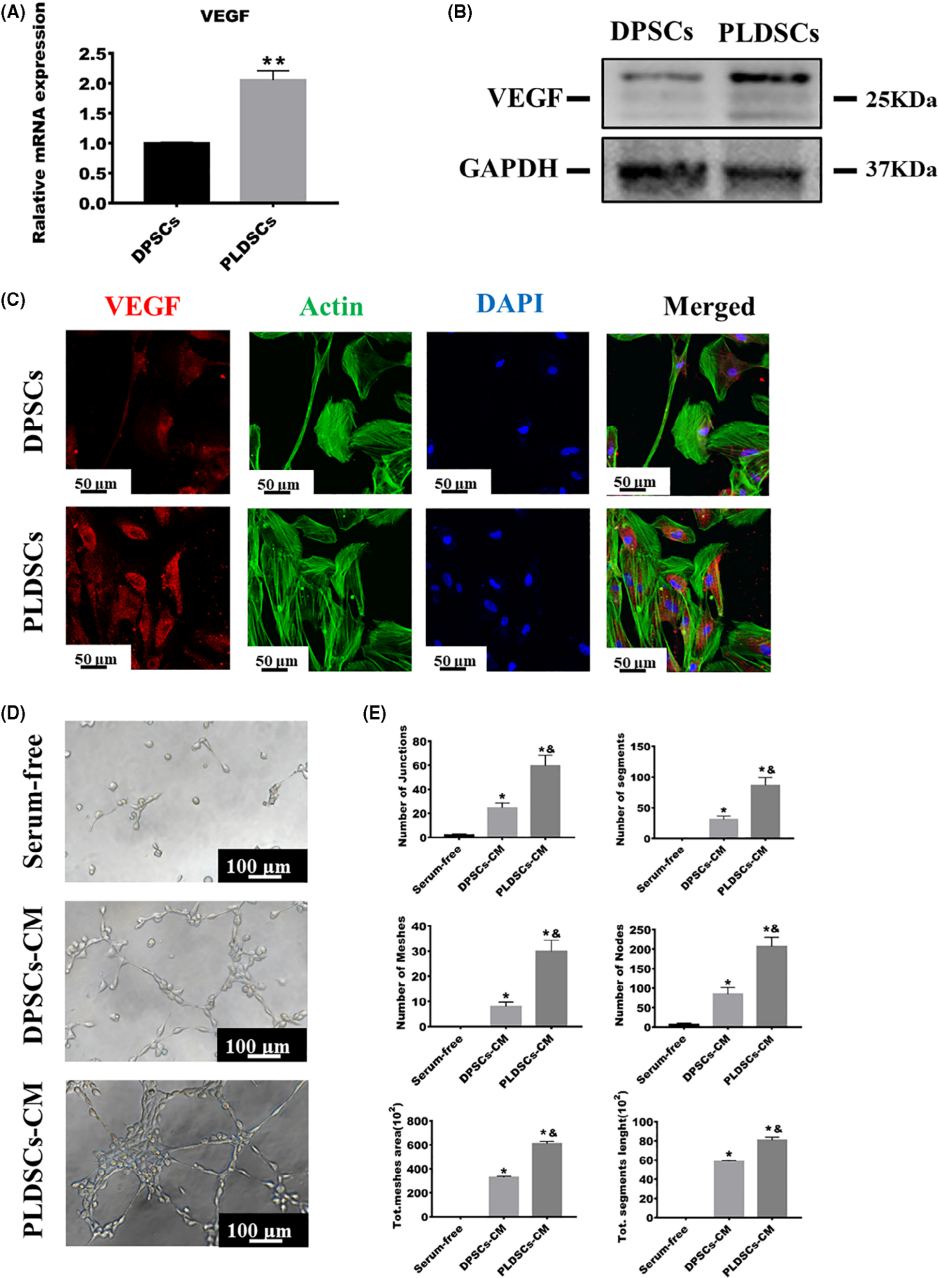
Comparison of pro‐angiogenesis properties of DPSCs and PLDSCs. (A) qRT‐PCR was used to compare the expression levels of *VEGF*; the expression levels were normalized to those of *ACTB* (*n* = 3, Student’s *t*‐test). **P < 0.01. (B) Western blotting detection of VEGF expression. (C) Immunofluorescence staining showing the protein expression of VEGF in the cytoplasm of DPSCs and PLDSCs. Scale bar = 50 μm. (D) Representative images for each group via inverted phase‐contrast microscopy. Scale bar = 100 μm. (E) Analysis of tube formation parameters (number of junctions, nodes, segments and meshes; total segment length; total mesh area) using imagej (*n* = 5, one‐way analysis of variance). *P < 0.05 with serum‐free, ^&^ *P* < 0.05 with DPSCs‐CM. DPSCs‐CM, dental pulp stem cells‐conditioned medium; PLDSCs‐CM, periapical lesion‐derived stem cells‐conditioned medium. Data are shown as the mean ± SD.

We next examined the pro‐angiogenic ability of the cells via qRT‐PCR analysis and found that the mRNA expression levels of VEGF were higher in PLDSCs than in DPSCs (Fig. 4A). Western blot results also showed that protein levels of VEGF were higher in PLDSCs (Fig. 4B). The results of immunofluorescence staining for VEGF were consistent with those of qRT‐PCR and western blotting; the fluorescence intensity for VEGF was higher in PLDSCs than in DPSCs (Fig. 4C). A tube formation assay was then performed to evaluate the pro‐angiogenic ability of different conditioned media on HUVECs. After 6 h, there was little tube formation in the control group. However, tubular structures were formed when HUVECs were incubated with DPSC‐ and PLDSC‐conditioned medium (Fig. 4D). Furthermore, analysis of various tube formation parameters, including the number of junctions, nodes, segments and meshes; total segment length; and total meshes area, as analyzed using imagej, indicated that PLDSCs‐conditioned medium was more effective for initiating angiogenesis (Fig. 4E).

### Neurotrophic ability of PLDSCs and DPSCs

**Fig. 5:**
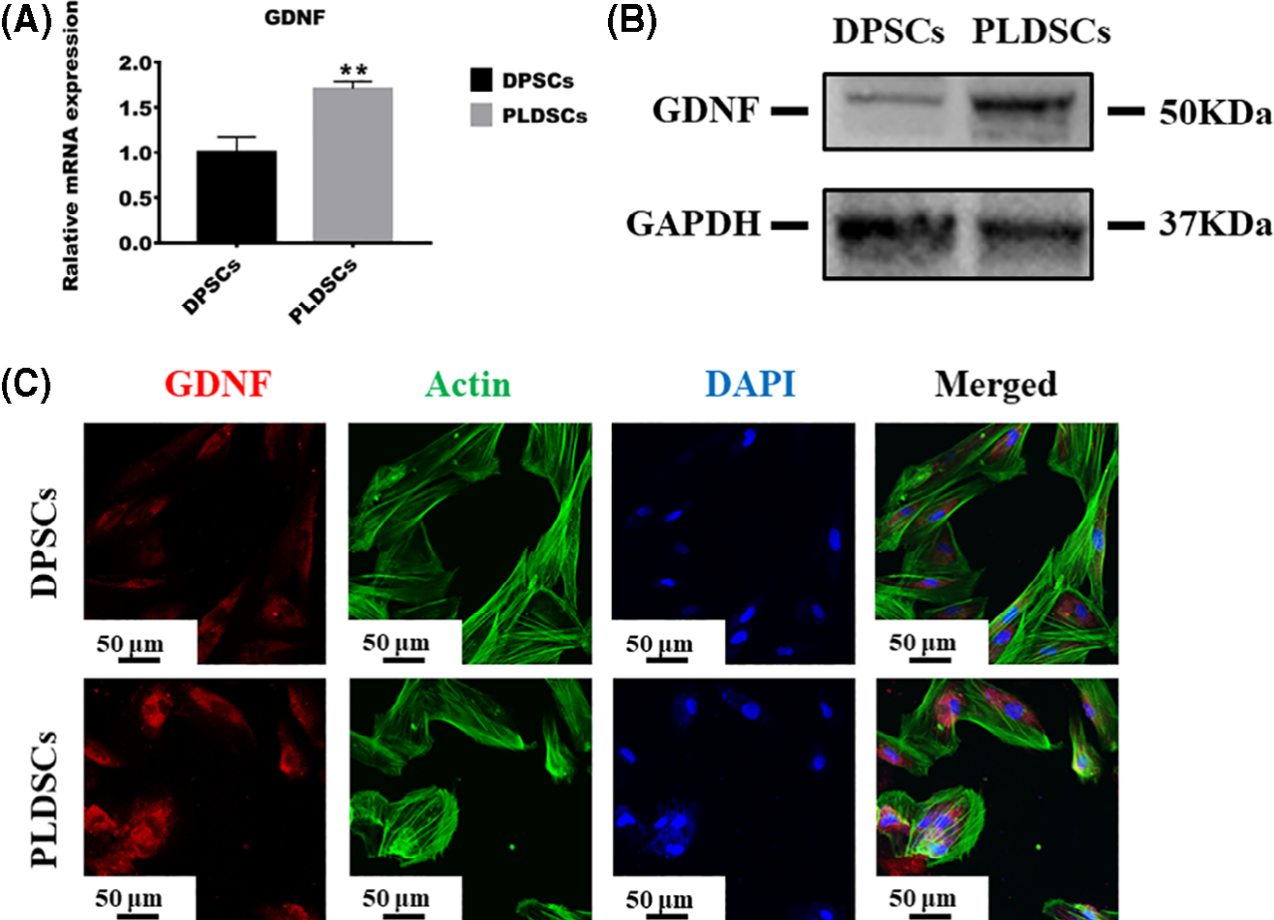
Comparison of neurotrophic ability of DPSCs and PLDSCs. (A) qRT‐PCR was used to compare the expression level of *GDNF* between PLDSCs and DPSCs; the expression level was normalized to that of *ACTB* (*n* = 3, Student’s *t*‐test). (B) Western blotting results of GDNF expression. (C) Immunofluorescence staining for GDNF expression in DPSCs and PLDSCs. Scale bar = 50 μm. **P < 0.01. Data are shown as the mean ± SD.

To evaluate neurotrophic ability, we examined the expression of GDNF, which is expressed in dental MSCs. The qRT‐PCR revealed the higher expression of GDNF in PLDSCs than in DPSCs (Fig. 5A). Western blot analysis showed that protein levels of GDNF were higher in PLDSCs (Fig. 5B). In addition, immunofluorescence staining showed a stronger fluorescence intensity for GDNF in PLDSCs than in DPSCs (Fig. 5C).

### Comparison of the immunomodulatory ability of the cells

**Fig. 6:**
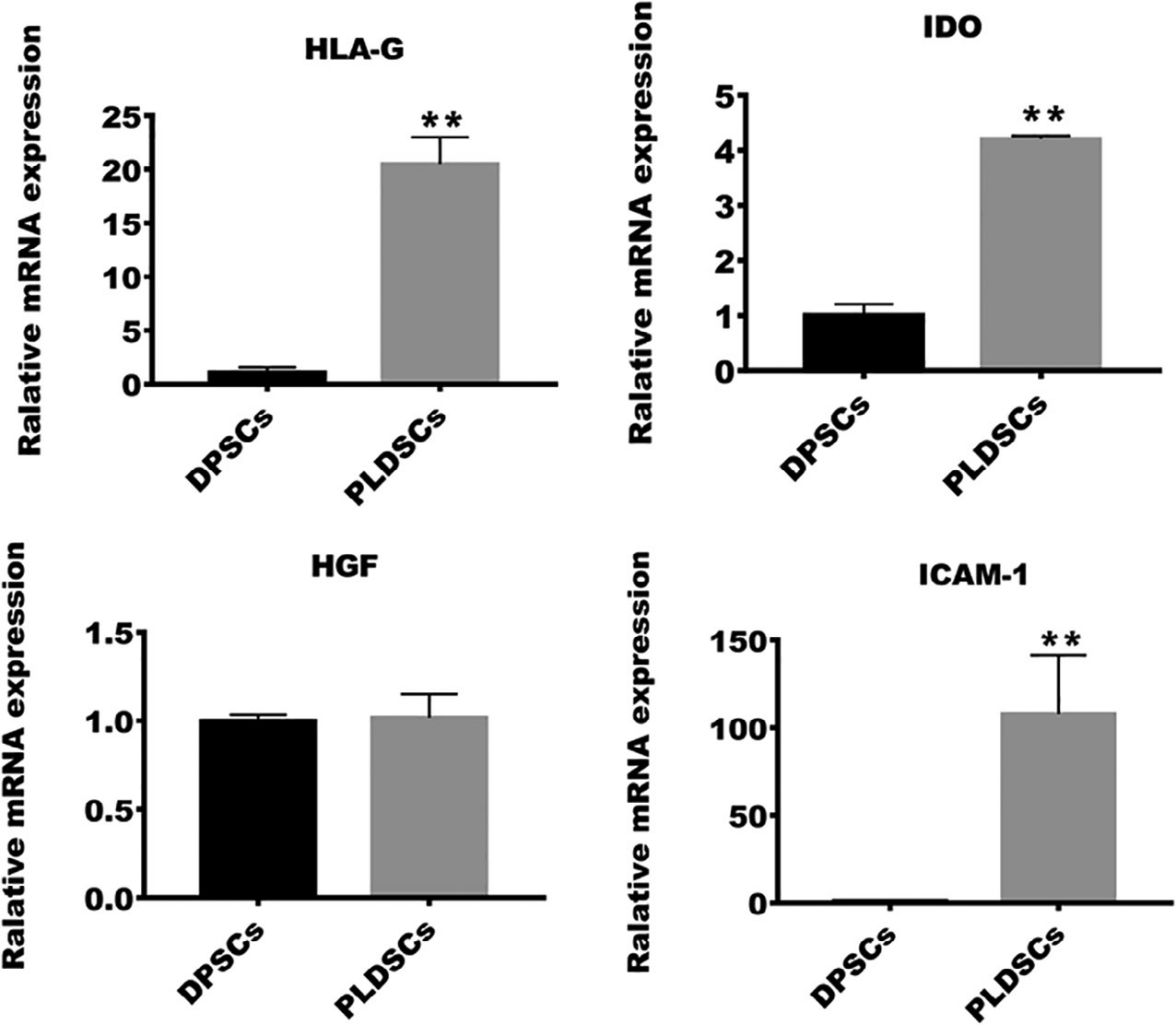
Comparison of the immunomodulatory abilities of the cells. Expression of immunomodulatory genes *HLA‐G*, *IDO*, *HGF* and *ICAM‐1* were evaluated via qRT‐PCR; *ACTB* was used for normalization (*n* = 3, Student’s *t*‐test). **P < 0.01. Data are shown as the mean ± SD.

We assessed the expression levels of immunomodulatory genes *IDO*, *HLA‐G*, *HGF* and *ICAM‐1* to compare the immunomodulatory abilities of the two cell types. PLDSCs expressed higher levels of *HLA‐G*, *IDO* and *ICAM‐1* than DPSCs. However, no significant difference was observed for expression of *HGF* (Fig. 6).

## Discussion

Derived from the mesoderm, dental MSCs are investigated intensely for their strong proliferation and adhesion abilities, as well as multilineage differentiation capacity [18]. However, well functioned dental MSCs are still difficult to mass produce to meet clinical demands and the properties vary under different circumstances. As a result, it is imperative to find more alternative seed cells for tissue engineering. Recently, periapical lesions were found to harbor cells exhibiting MSC‐like properties, suggesting that the use of these pathological tissues should be re‐evaluated [12, 17, 19, 20, 21]. However, the MSCs isolated from periapical lesions had not been investigated from the viewpoint of dental pulp regeneration. Therefore, we aimed to more closely examine the properties of PLDSCs by comparing them with DPSCs, the most widely used seed cells for dental pulp tissue regeneration to date [22].

Using a tissue outgrowth method, the present study obtained spindle‐like and plastic‐adhesive cells from periapical lesions. Under different conditioned media, these types of cell could achieve osteogenic, adipogenic and chondrogenic differentiation. In addition, flow cytometry results showed that PLDSCs were positive for CD29, CD105, CD44, CD73 and CD90 and negative for hematopoietic markers CD31, CD34 and CD45, which was similar to that for DPSCs. However, it was also reported that the different culture methods and passage numbers will alter the properties and marker profile of MSCs [23, 24]. Therefore, to acquire an objective baseline, we used passage 3 for both cell types when conducting our experiments.

For the purposes of stem cell therapy and tissue regeneration, which require a tremendous number of cells, *in vitro* cell proliferation ability is of great importance [25]. It is well known that dental tissue‐derived MSCs, particularly DPSCs, have strong proliferation ability [25]. In the present study, we found similar results indicating that DPSCs had stronger proliferative ability [17], with the proliferation rate of DPSCs being 2.07 ± 0.04‐fold compared to that of PLDSCs on day 11. Given that the CCK‐8 assay is an indirect method for evaluating proliferation, we then performed a CFU‐F assay. The results of the CFU‐F assay showed that PLDSCs had a self‐renewal ability similar to that of DPSCs. Taken together, PLDSCs could be cultured and actively proliferate *in vitro*. Furthermore, the migration ability of cells should be taken into consideration because injured tissues secrete chemokines to attract stem cells from other sites and initiate tissue repair. In the present study, we showed that PLDSCs were more active and had higher migration ability than DPSCs. This finding may be explained by the distinct environment of PLDSCs, where many pro‐inflammatory cytokines are present. For example, the pro‐inflammatory chemokine RANTES/CCL5 has been reported to increase the migration ability of periodontal ligament stem cells isolated from inflamed periodontal ligaments [26].

For the regeneration of the dentin–pulp complex, seed cells must be capable of osteo/odontogenic differentiation. It was reported that different isolation methods, culture environment, commercial osteo/odontogenic induced medium and inflammatory stages will also lead to different osteo/odontogenesis results [23, 27, 28]. In the present study, we isolated MSCs by the outgrowth method and found that PLDSCs showed weaker osteo/odontogenic differentiation capacities than DPSCs, which is consistent with the results for alveolar bone resorption symptoms in patients with periapical periodontitis. Similarly, inflammation was reported to compromise the osteo/odontogenic capacity of MSCs. Lee *et al*. [29] reported that DPSCs from inflamed dental pulp may have decreased osteo/odontogenic capacity compatred to DPSCs from normal dental pulp. *In vitro* studies using *Pg* lipopolysaccharide or tumor necrosis factor‐α to simulate the inflammatory environment also showed the decreased osteo/odontogenic ability of dental MSCs [30, 31]. By contrast, a comparative study using an enzyme digestion method to isolate MSCs showed that PLDSCs had stronger osteogenic differentiation ability than DPSCs and dental follicle stem cells [17]. Other studies applying *Escherichia coli* lipopolysaccharide or a mixture of pro‐inflammatory cytokines to simulate inflammatory conditions *in vitro* have demonstrated increased ALP activity and calcification ability in bone marrow stem cells, adipose stem cells and DPSCs [11, 32]. The controversy regarding inflammation and the osteo/odontogenic capacity of MSCs may be explained by the complexity of the inflammatory niche, which contains various cytokines and immune cells. Therefore, to achieve better osteo/odontogenesis of PLDSCs, further studies are needed to investigate the potential regulatory cytokines or immune cells in the different stages of periapical lesions.

It is widely accepted that oxygen can only diffuse approximately 200 μm through tissues [33]; therefore, sufficient vessel networks are vital for the survival of engineered regenerative tissues; this is particularly true for dental pulp, which only has a small opening (< 1 mm) for blood vessels to enter [14]. The incorporation of growth factors or the co‐culture of endothelial cells with pro‐angiogenic cells are the two major approaches used to solve this problem [22]. Among the various grow factors, VEGF can be secreted by dental MSCs in a paracrine way [15] and has a strong impact on blood vessel initiation, being effective for enhancing vessel density in the dental pulp [34]. Yet, no research has focused on the pro‐angiogenesis potential of PLDSCs. In the present study, the secretion of VEGF by DPSCs and PLDSCs was confirmed via qRT‐PCR, western blotting and immunofluorescence; we observed that VEGF expression was higher in PLDSCs than in DPSCs. It has been reported that different stress conditions will give rise to different secretions and concentrations of pro‐angiogenesis growth factors [35]. Of these conditions, hypoxia is considered to be a common driving force for the dramatic increase in VEGF expression observed in injured and inflamed dental pulp [36]. In response to hypoxia, dental pulp cells increase the expression of hypoxia‐inducible factor 1 and then mediate the increased transcription of pro‐angiogenesis factors, including VEGF, platelet‐derived growth factor AB and angiopoietin [14]. A recent study sequencing RNA from 10 periapical periodontitis tissues also revealed that the hypoxia‐inducible factor 1 pathway was activated [37]. Therefore, it is reasonable to assume that the PLDSCs in the present study expressed higher levels of VEGF and that PLDSC‐conditioned medium induced increased tubular structure formation in HUVECs because the PLDSCs resided in a hypoxic inflammatory condition before surgery. This enhanced pro‐angiogenic ability is promising with respect to addressing insufficient angiogenesis in dental pulp engineering.

Neurogenesis is essential for functional pulp regeneration. Nerves in the pulp chamber help adjust the masticatory force when chewing and nourish blood vessels inside the pulp by secreting multiple growth factors. DPSCs are able to differentiate into neural lineage and secret neurotrophic factors, being promising in neural repair and regenerative in nerve diseases [38]. PLDSCs were also capable of differentiating into neuronal cells [13]. In the present study, we chose GDNF to evaluate its neurotrophic function. GDNF is an important neurotrophin involved in the survival of neurons, differentiation of neuroblasts and neuritogenesis [39]. Furthermore, it was reported that GDNF was able to exert anti‐pro‐inflammation function in renal interstitial fibrosis and inflammatory bowel disease [40, 41]. GDNF upregulation was found in lipopolysaccharide‐induced nigral inflammation [42]. In the present study, we showed similar results indicating that PLDSCs expressed higher GDNF levels than DPSCs, suggesting the potential use of PLDSCs in regenerating dental pulp nerves, or even in other nerve diseases [43, 44].

Although autologous stem cell‐based tissue engineering is the most ideal approach for tissue regeneration, it is not suitable for all patients because the self‐renewal and differentiation abilities of stem cells depend on the health status of the host. Therefore, an allogenic stem cell‐based approach is still an alternative, and an immunomodulatory effect should also be considered when applying seed cells. MSCs are able to secret immunomodulatory factors and suppress immunocytes directly via a cell–cell interaction [45]. Indoleamine 2,3‐dioxygenase (IDO), human leukocyte antigen G (HLA‐G), hepatocyte growth factor (HGF) and intercellular adhesive molecule‐1 (ICAM‐1) have been implicated in MSC‐mediated immunomodulation. IDO is an enzyme that converts tryptophan into kynurenine and leads to anergy in dendritic cells and T cells [46]. DPSCs from inflamed pulp have been shown to exhibit increased IDO expression compared to DPSCs from healthy pulp [47]. Another crucial cytokine, HLA‐G, is a nonclassical MHC class I involved in immunomodulation by inhibiting the activation of natural killer cells and effector T cells [48, 49]. A previous study reported that simulating the inflammatory environment of dental MSCs by incubating the cells with the pro‐inflammatory cytokine interferon‐γ results in increased HLA‐G expression in an interferon‐γ‐dependent manner [50]. Co‐culture experiments with peripheral blood mononuclear cells also drive the increased expression of IDO, soluble HLA‐G and HGF in MSCs [51, 52]. ICAM‐1 also functions in MSC‐mediated immunomodulation by inhibiting DC maturation and the T‐cell response [53, 54]. Studies have shown the improved immunosuppressive ability of MSCs with increased ICAM‐1 expression; however, this effect was impaired when ICAM‐1 was inhibited or knocked down in MSCs [55, 56]. PLDSCs was also reported to inhibit proliferation, function and pro‐inflammation cytokines secretion of immune cells [17, 57]. Yet, whether this inflammatory niche of periapical lesions alters the immunomodulatory ability of PLDSCs remains unknown. In the present study, the expression levels of *IDO*, *HLA‐G* and *ICAM‐1* were higher in PLDSCs than in DPSCs, suggesting that the inflammatory environment of periapical lesions is beneficial for the immunomodulatory ability of PLDSCs and that this ability could be retained after culturing *in vitro*. This property of PLDSCs may be utilized in allogenic stem cell therapy or allogenic stem cell‐based dental pulp tissue engineering to achieve a weak graft versus host response.

In summary, the present study demonstrates that PLDSCs might be optional seed cells for dental pulp regeneration, especially for dental neurovascularization with less ethical problems. As shown in Table 2, PLDSCs can actively proliferate *in vitro* and have weaker osteo/odontogenic ability, but have stronger migration, pro‐angiogenesis, neurotrophic and immunomodulatory abilities, compared to DPSCs. Therefore, they have promising application in autologous or allogenic stem cell‐based dental pulp regeneration. Beyond their application in dental disorders, they are also promising with respect to neurological disease [43]. However, for partial pulpectomy investigations with the aim of regenerating only dentin, DPSCs are recommended. Accordingly, the osteo/odontogenic differentiation ability of PLDSCs and correlations with different types of periapical lesions require more investigation. Furthermore, additional studies are warranted aiming to elucidate the mechanisms regulating the modified neurovascular induction and immunomodulatory abilities of these cells, their potential risk of tumorigenesis, and their *in vivo* effects.

**Table 2:** Summary of the properties of DPSCs and PLDSCs

	DPSCs	PLDSCs
Morphology	Spindle‐like shape	
Immunophenotype	CD29(+)CD105(+)CD44(+)CD73(+)CD90(+)/CD31(–)CD34(–)CD45(–)	
Adipogenic and chondrogenic differentiation	No obvious differences	
Proliferation	Stronger	
Migration		Stronger
Osteo/odontogenesis	Stronger	
Pro‐angiogenesis		Stronger
Neurotrophic ability		Stronger
Immunomodulatory ability		Stronger

## Conflict of interests

The authors declare that they have no conflicts of interest.

## Author contributions

WPL and MYM conducted most of the experiments and drafted the manuscript. NH contributed to the collection of clinical samples and data analysis, and also revised the manuscript. JW and JH revised the manuscript. SSG contributed to study conception and design and critically revised the manuscript. All authors read and approved the final manuscript submitted for publication.

## Data Availability

All of the data generated or analyzed during the present study are included in this article.
